# Oral Infection

**Published:** 1921-06

**Authors:** A. W. Thornton


### ORAL INFECTION

I do not know how you would make a fixed bridge if it was for the posterior part of the mouth. What we call sanitary bridges, which do not touch the alveolar crest, may be made so that a piece of rag may be drawn from one side to the other and the cap polished in that way. But certainly, fixed bridges as they are made and as we see them in the mouths of patients are not cleanly, and I think the general consensus of opinion will endorse that position.

Dr. Nolin said during the course of his remarks that there were cases in which a fixed bridge possibly saved a man's life. I can not conceive of any such case as that. I wish it had been possible for you to see two removable bridges which were in the mouth of one of the members who was in attendance at this convention, but who had to go home last night. He had lost a lower molar on each side—a first molar or second molar on each side. Which one of you would have the tooth anterior and posterior to the vacant space ground down in order to supply that one tooth? Yet this man is one of the most scientific men in the country. He realized that his teeth were drifting a little and producing traumatic occlusion, so he constructed for himself two removable bridges along the line of the technique suggested by Dr. Nesbitt, of Boston. I said to him within the last seven days: "How long did it take you to become accustomed to them—until they were comfortable?" He said: "They were comfortable from the time I first put them in; I am not conscious that they are there; I bite on them as comfortably as possible." Now, is that not better practice—to put in removable pieces that can be taken out and cleaned, without the destruction of any serviceable tooth tissue? Is that not better than putting in the best kind of fixed bridgework? I believe it is. If you have a missing lateral in the anterior part of the mouth, and you can make one of those half-crowns, casting it with a shoulder somewhat along the line of the Carmichael attachment, I believe that a fixed piece can be kept clean there because there is no occlusal surface involved. But in the posterior part of the mouth I say it is next to impossible to do that. I can not conceive of a place where it would not be better to put in a removable bridge than a fixed bridge. Let me say that we owe this to the inception of bridgework; that it has brought about a perfection of technique which, perhaps, we should never have had but for the advent of fixed bridgework. It brought with it another advantage: that men in the dental profession commenced to get a fee commensurate with the service which they rendered. The time has now come when the members of the profession can get just as good a fee and adopt a better plan of practice. The public today recognize the fact that dentistry is necessary and that it should be paid for. At first, bridgework commanded a high fee because so few men were doing it; and the size of the fee made the wealthy people think that they were getting superior service.

Most of you know Dr. Arthur Black; he is not a man who could be easily imposed upon. Three years ago I was attending a meeting in Pittsburgh. In company with Dr. Black, I was walking to the institute where they do the research work. He opened his mouth and said: ''How do you like my bridge?'' I could not see any bridge, but he had one in his mouth. This was its history: both upper centrals had become devitalized through apical abscesses. His father treated them; one yielded to treatment, but the other did not. Other men gave him treatment, but still the abscess did not heal. Finally the tooth was extracted; and he put a bar of gold on the lingual side and attached it to the other devitalized tooth so that he had his own tooth as a bridge. ''For years,'' he said, ''I have had in the sole of my foot a little boil. Pus would accumulate there; I would open it, and it would give me no more discomfort for a time; then perhaps in three or four weeks it would come again. That has been going on for years, but since I have had this tooth extracted and the socket curetted, this trouble has disappeared.'' A few months ago I was at a meeting of the Pennsylvania Society in Pittsburgh, and Black was there. I said: ''How about your bridge and how about the boil in the sole of your foot?'' He said: ''I had one recurrence of it about three weeks afterwards, and I have never been troubled with it since.'' Now, he is not a quack; he is a man who is able to draw a fairly logical conclusion.

I do not take the position that I do because I am a better operator than any one else. Some one has referred to standing or distinction or something of that kind. Well, let there be no more of that so far as I am concerned, for I am one of the very humblest of men. But I have had a chance to observe the results following from mouth infection. I have seen deaths occur from this cause. I have seen scores of people who were crippled, and I have seen many of these restored to health by the extraction of teeth and the curetting of sockets. I know what I am talking about when I say that it is one of the hardest things any man can undertake in the mechanical line to make a crown that will fit under the free margin of the gum. If you make such crowns at all, do not let them touch the free margin of the gum.

If you give this matter serious thought, you will come to the conclusion that there is no case in which you can not do yourself and your patient better justice by putting in removable appliances—and not, by the way, one of Shea's appliances; for it requires the destruction of too much tissue; it is complicated, and when it breaks it is hard to repair. I have been in Shea's place many times; I know him very well.

You talk about the man who five years ago said one thing and today says another. Well, is that not as it should be? Surely a man should not stagnate. Up in Ontario, where everything is right, a judge was trying a case. He committed it to the jury, and after three hours the jury returned and the judge said: "Are you agreed on a verdict?" The foreman said: "No, my lord, we are not." Said the judge: "Is there any possibility of agreement?" The foreman replied: "I do not know; we stand eleven to one." The judge said: "Well, I am going to send you back. I have no right to dictate to you what your finding shall be in this case, but I want to direct the attention of the one man to the fact that a wise man may change his opinion, but that a fool never does." In the light of our present knowledge, with the information that we get through the use of X-rays; with what we know of pathology and of selective capacity and of pathogenic organisms, would we not be fools if we held to the same opinions today that we held when Foster Flagg defended the filling of roots with cotton on the ground that experiments with a test tube containing contaminated material proved that the cotton prevented the ingress of organisms? But that absorbent cotton was not in contact with the contaminated material in the test tube; and we know today that if you put cotton and creosote in a tooth in contact with putrescent matter, it is not very long before the cotton itself is contaminated also. So we are going on from grace to grace and from glory to glory—that is more Scripture!—Dr. A. W. Thornton, in *Oral Health.*

